# Complete genome sequence of *Geodermatophilus obscurus* type strain (G-20^T^)

**DOI:** 10.4056/sigs.711311

**Published:** 2010-03-30

**Authors:** Natalia Ivanova, Johannes Sikorski, Marlen Jando, Christine Munk, Alla Lapidus, Tijana Glavina Del Rio, Alex Copeland, Hope Tice, Jan-Fang Cheng, Susan Lucas, Feng Chen, Matt Nolan, David Bruce, Lynne Goodwin, Sam Pitluck, Konstantinos Mavromatis, Natalia Mikhailova, Amrita Pati, Amy Chen, Krishna Palaniappan, Miriam Land, Loren Hauser, Yun-Juan Chang, Cynthia D. Jeffries, Linda Meincke, Thomas Brettin, John C. Detter, Manfred Rohde, Markus Göker, Jim Bristow, Jonathan A. Eisen, Victor Markowitz, Philip Hugenholtz, Nikos C. Kyrpides, Hans-Peter Klenk

**Affiliations:** 1 DOE Joint Genome Institute, Walnut Creek, California, USA; 2 DSMZ – German Collection of Microorganisms and Cell Cultures GmbH, Braunschweig, Germany; 3 Los Alamos National Laboratory, Bioscience Division, Los Alamos, New Mexico, USA; 4 Biological Data Management and Technology Center, Lawrence Berkeley National Laboratory, Berkeley, California, USA; 5 Oak Ridge National Laboratory, Oak Ridge, Tennessee, USA; 6 Lawrence Livermore National Laboratory, Livermore, California, USA; 7 HZI – Helmholtz Centre for Infection Research, Braunschweig, Germany; 8 University of California Davis Genome Center, Davis, California, USA

**Keywords:** aerobic, non-pathogenic, soil and rock varnish, morphogenetic growth cycle of C-form and R-form, *Frankineae*, *Actinobacteria*, GEBA

## Abstract

*Geodermatophilus obscurus* Luedemann 1968 is the type species of the genus, which is the type genus of the family *Geodermatophilaceae*. *G. obscurus* is of interest as it has frequently been isolated from stressful environments such as rock varnish in deserts, and as it exhibits interesting phenotypes such as lytic capability of yeast cell walls, UV-C resistance, strong production of extracellular functional amyloid (FuBA) and manganese oxidation. This is the first completed genome sequence of the family *Geodermatophilaceae*. The 5,322,497 bp long genome with its 5,161 protein-coding and 58 RNA genes is part of the *** G****enomic* *** E****ncyclopedia of* *** B****acteria and* *** A****rchaea * project.

## Introduction

Strain G-20^T^ (= DSM 43160 = ATCC 25078 = JCM 3152) is the type strain of the species *Geodermatophilus obscurus*, which is the type genus in the family *Geodermatophilaceae* [1,2]. The species name derives from the Latin word ‘obscurus’ meaning dark, obscure, indistinct, unintelligible [1]. The genus *Geodermatophilus* and family *Geodermatophilaceae* were originally proposed in 1968 by Luedemann [1]. The genus *Geodermatophilus* was first described as a genus closely related to genus *Dermatophilus*, but being isolated from soil, as indicated by the prefix ‘geo’, which derives from Greek ‘Gea’ meaning Earth [1]. In contrast, members of the genus *Dermatophilus* originated from skin lesions of cattle, sheep, horses, deer, and man [3], as the meaning of the genus name is ‘skin-loving’. Yet, on the basis of 16S rRNA gene sequences, *Geodermatophilus* proved to be only distantly related to *Dermatophilus* [4] and was thus included in 1989 in the family *Frankiaceae* [5], together with the genera *Blastococcus* and *Frankia*. In 1996, the genera *Dermatophilus* and *Blastococcus* were excluded again from the family *Frankiaceae* [6] and finally formally combined with the genus *Modestobacter* in the family *Geodermatophilaceae* again [2]. *G. obscurus* is the only validly described species in the genus *Geodermatophilus* [7], and consists of four subspecies [1] which have never been validly published [8].

The type strain G-20^T^, together with other strains, has been isolated from soil in the Amargosa Desert of Nevada, USA [3]. Further *Geodermatophilus* strains were isolated from limestone [8,9] and rock varnish [10] in the Negev Desert, Israel, from marble in Delos, Greece [8,9], from chestnut soil in Gardabani, Central Georgia [11], from rock varnish in the Whipple Mountains, California, USA [12], from orange patina of calcarenite in Noto, Italy [13], from gray to black patinas on marble in Ephesus, Turkey [13], and from high altitude Mount Everest soils [14,15]. Here we present a summary classification and a set of features for *G. obscurus* G-20^T^, together with the description of the complete genomic sequencing and annotation.

## Classification and features

**Figure 1:**
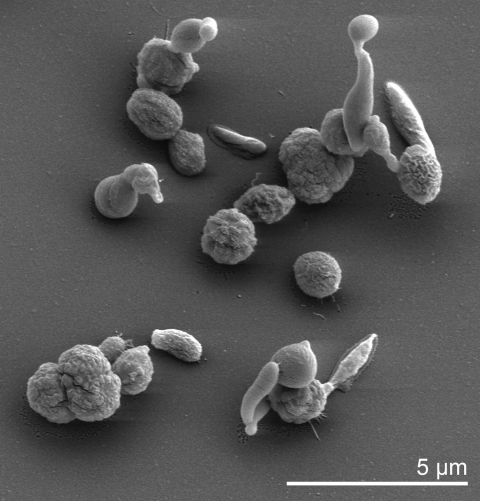
Scanning electron micrograph of *G. obscurus* G-20^T^

Cells of *Geodermatophilus* produce densely packed cell aggregates [8], which are described as a muriform, tuber-shaped, noncapsulated, holocarpic thallus consisting of masses of cuboid cells averaging 0.5 to 2.0 µm in diameter (Table 1 and Figure 1) [1]. The thallus breaks up, liberating cuboid or coccoid nonmotile cells and elliptical to lanceolate zoospores [1]. The single cell can differentiate further into polar flagellated motile zoospores [15]. Thus, cells of *Dermatophilus* may express a morphogenetic growth cycle in which it switches between a thalloid C-form and a motile zoosporic R-form [15]. It has been supposed that tryptose (Difco) contains an unidentified factor, M, which controls morphogenesis in *Geodermatophilus* [15], though others could not observe the motile, budding zoospores of the R-form [8]. As colonies, strains of *Geodermatophilus* strains exhibit usually a dark brownish, greenish, or black pigmentation with a smooth to rough surface and in most cases a solid consistency, including minor variations in colony shape [8]. Young colonies are almost colorless, having smooth edges which become distorted and lobed in older colonies, where the colony consistency becomes somewhat crumby [8]. The colonies become darkly pigmented immediately when they started to protrude upwards in the space above the agar [8]. *Geodermatophilus* does not produce hyphae, vesicles, outer membranous spore layers or capsules [5].

**Table 1:** Classification and general features of *G. obscurus* G-20^T^ according to the MIGS recommendations [16]

**MIGS ID**	**Property**	**Term**	**Evidence code**
	Current classification	Domain *Bacteria*	TAS [17]
		Phylum *Actinobacteria*	TAS [18]
		Class *Actinobacteria*	TAS [19]
		Subclass *Actinobacteridae*	TAS [19]
		Order *Actinomycetales*	TAS [19]
		Suborder *Frankineae*	TAS [19]
		Family *Geodermatophilaceae*	TAS [2]
		Genus *Geodermatophilus*	TAS [1]
		Species *Geodermatophilus obscurus*	TAS [1]
		Type strain G-20	TAS [1]
	Gram stain	gram positive	TAS [1]
	Cell shape	cuboid or coccoid nonmotile cells and elliptical to lanceolate zoospores	TAS [1]
	Motility	motile zoospores	TAS [1]
	Sporulation	unknown	TAS [1]
	Temperature range	18°C–37°C	TAS [20]
	Optimum temperature	24°C-28°C	TAS [20]
	Salinity	does not grow at 3% or more NaCl	TAS [20]
MIGS-22	Oxygen requirement	aerobic	TAS [20]
	Carbon source	soluble sugars	TAS [1]
	Energy source	chemoorganotroph	TAS [8]
MIGS-6	Habitat	worldwide distribution in soil, on rock surfaces, and deep sea marine sediments	TAS [2,8]
MIGS-15	Biotic relationship	free-living	TAS [1,8,10,12,14]
MIGS-14	Pathogenicity	no	NAS
	Biosafety level	1	TAS [21]
	Isolation	soil	TAS [1]
MIGS-4	Geographic location	Amargosa Desert, Nevada, USA	TAS [1]
MIGS-5	Sample collection time	1968, or before	TAS [1]
MIGS-4.1 MIGS-4.2	Latitude Longitude	36.48 -116.50	NAS
MIGS-4.3	Depth	unknown	
MIGS-4.4	Altitude	unknown	

Evidence codes - IDA: Inferred from Direct Assay (first time in publication); TAS: Traceable Author Statement (i.e., a direct report exists in the literature); NAS: Non-traceable Author Statement (i.e., not directly observed for the living, isolated sample, but based on a generally accepted property for the species, or anecdotal evidence). These evidence codes are from of the Gene Ontology project [22]. If the evidence code is IDA, then the property was directly observed by one of the authors or an expert mentioned in the acknowledgements.

Strain G-20^T^ utilizes L-arabinose, D-galactose, D-glucose, glycerol, inositol, D-levulose, D-mannitol, sucrose, and D-xylose as single carbon sources for growth, but not D-arabinose, dulcitol, β-lactose, melezitose, α-melibiose, raffinose, D-ribose, and ethanol [1,23]. Growth with L-rhamnose is only poor [1]. Strain G-20^T^ is negative for β-hemolysis of blood agar (10% human blood) [1]. Also, nitrate reduction occurs only sporadically with both inorganic or organic nitrate broth [1]. Strain G-20^T^ hydrolyses starch, is weakly positive for gelatin liquefaction and negative for casein utilization [23].

Strain G-20^T^ showed a remarkable production of extracellular functional bacterial amyloid (FuBA), which is accessible to WO2 antibodies without saponification [24]. The WO2 antibody has been shown to bind only to amyloid and not to other kinds of protein aggregates [20,24]. One strain of *G. obscurus* was described as having a lytic activity on yeast cell walls [12]. Another strain from rock varnish was shown to exhibit very strong resistance to UV-C light (220 J×m^-2^) [12]. Two strains from rock varnish in the Negev Desert were able to oxidize manganese [10].

Only three *G. obscurus* isolates have 16S rRNA gene sequences with >98% sequence similarity to strain G-20^T^: isolate G18 from Namibia, 99.1% [2], isolate 06102S3-1 from deep-sea sediments of the East Pacific and Indian Ocean (EU603760) 98.5%, and *G. obscurus* subspecies *utahensis* DSM 43162, 98.03% [8]. The highest degree of sequence similarity in environmental metagenomic surveys, 93.3% was reported from a marine metagenome (AACY020064011) from the Sargasso Sea [25]. (January 2010).

**Figure 2:**
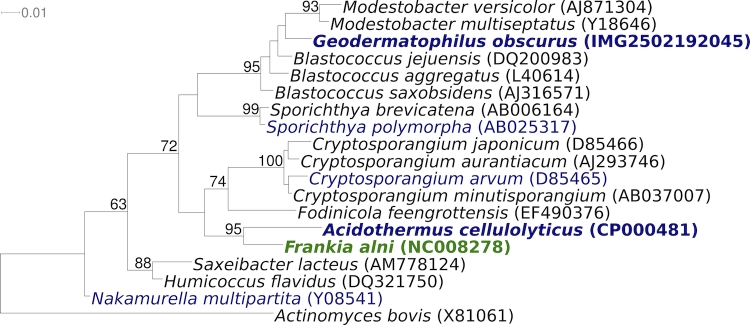
Phylogenetic tree highlighting the position of *G. obscurus* G-20^T^ relative to the other type strains within the suborder *Frankineae*. The tree was inferred from 1,364 aligned characters [26,27] of the 16S rRNA gene sequence under the maximum likelihood criterion [28] and rooted with the type strain of the order *Actinomycetales*. The branches are scaled in terms of the expected number of substitutions per site. Numbers above branches are support values from 350 bootstrap replicates [29] if larger than 60%. Lineages with type strain genome sequencing projects registered in GOLD [30], such as the GEBA organism *Nakamurella multipartita* [31] are shown in blue. Important non-type strains are shown in green [32], and published genomes in bold.

Figure 2 shows the phylogenetic neighborhood of for *G. obscurus* G-20^T^ in a 16S rRNA based tree. The sequences of the three 16S rRNA gene copies in the genome of *G. obscurus* G-20^T^ do not differ from each other, but differ by 24 nucleotides from the previously published 16S rRNA sequence obtained from DSM 43160 (X92356). These considerable discrepancies are most likely due to sequencing errors in the latter sequence. Genbank accession L40620, which was obtained from ATCC 25078, differs by only one single nucleotide from the 16S rRNA gene copies in the genome obtained from DSM 43160.

### Chemotaxonomy

The major fatty acids of strain G-20^T^ are iso-C_15:0_ (19.0%), iso-C_16:0_ (16.2%), C_16:1 cis9_ (13:0%), C_17:1_ (10.4%), C_18:1 cis9_ (6.6%), and anteiso-C_15:0_ (5.7%). All other fatty acids (iso-C_14:0_, C_15:0_, C_15:1_, C_16:0_, C_17:0_, iso-C_17:0_, and anteiso-C_17:0_) were each below 4% [33]. Qualitatively, these values are largely congruent with other sources [4,34]. Strain G-20^T^ contains tetrahydro-menaquinones with nine isoprene units [MK-9(H_4_)] as sole component [4]. No whole cell wall sugar was found in strain G-20^T^, which contains only small amounts of galactose, glucose, and ribose [4,35]. The cell wall type is IIIC, and contains meso-2,6-diaminopimelic acid [35].

## Genome sequencing and annotation

### Genome project history

This organism was selected for sequencing on the basis of its phylogenetic position, and is part of the *** G****enomic* *** E****ncyclopedia of* *** B****acteria and* *** A****rchaea * project. The genome project is deposited in the Genome OnLine Database [30] and the complete genome sequence is deposited in GenBank. Sequencing, finishing and annotation were performed by the DOE Joint Genome Institute (JGI). A summary of the project information is shown in Table 2.

**Table 2:** Genome sequencing project information

**MIGS ID**	**Property**	**Term**
MIGS-31	Finishing quality	Finished
MIGS-28	Libraries used	One 8kb pMCL200 genomic library One 454 pyrosequencing standard library and one Illumina library
MIGS-29	Sequencing platforms	ABI3730, 454 GS FLX, Illumina GA
MIGS-31.2	Sequencing coverage	8.0× Sanger; 21.8× pyrosequencing
MIGS-30	Assemblers	Newbler version 1.1.02.15, phrap
MIGS-32	Gene calling method	Prodigal, GenePRIMP
	INSDC ID	CP001867
	Genbank date of release	January 19, 2010
	GOLD ID	Gc01190
	NCBI project ID	29547
	Database: IMG-GEBA	2502171147
MIGS-13	Source material identifier	DSM 43160
	Project relevance	Tree of Life, GEBA

### Growth conditions and DNA isolation

*G. obscurus* G-20^T^, DSM 43160, was grown in DSMZ medium 65 [36] at 28°C. DNA was isolated from 0.5-1 g of cell paste using Qiagen Genomic 500 DNA Kit (Qiagen, Hilden, Germany) with a modified protocol for cell lysis, (procedure st/L), and one hour incubation at 37°C, according to Wu *et al*. [37].

### Genome sequencing and assembly

The genome was sequenced using a combination of Sanger and 454 sequencing platforms. All general aspects of library construction and sequencing performed at the JGI can be found at the JGI website (http://www.jgi.doe.gov/). 454 Pyrosequencing reads were assembled using the Newbler assembler version 1.1.02.15 (Roche). Large Newbler contigs were broken into 5,725 overlapping fragments of 1,000 bp and entered into assembly as pseudo-reads. The sequences were assigned quality scores based on Newbler consensus q-scores with modifications to account for overlap redundancy and adjust inflated q-scores. A hybrid 454/Sanger assembly was made using the parallel phrap assembler (High Performance Software, LLC). Possible misassemblies were corrected with Dupfinisher or transposon bombing of bridging clones [38]. A total of 1,530 Sanger finishing reads were produced to close gaps, to resolve repetitive regions, and to raise the quality of the finished sequence. Illumina reads were used to improve the final consensus quality using an in-house developed tool (the Polisher). The error rate of the completed genome sequence is less than 1 in 100,000. Together, the combination of the Sanger and 454 sequencing platforms provided 29.8× coverage of the genome. The final assembly contains 48,209 Sanger reads and 353,553 pyrosequencing reads.

### Genome annotation

Genes were identified using Prodigal [39] as part of the Oak Ridge National Laboratory genome annotation pipeline, followed by a round of manual curation using the JGI GenePRIMP pipeline [40]. The predicted CDSs were translated and used to search the National Center for Biotechnology Information (NCBI) nonredundant database, UniProt, TIGR-Fam, Pfam, PRIAM, KEGG, COG, and InterPro databases. Additional gene prediction analysis and functional annotation was performed within the Integrated Microbial Genomes - Expert Review (IMG-ER) platform [41].

## Genome properties

**Figure 3:**
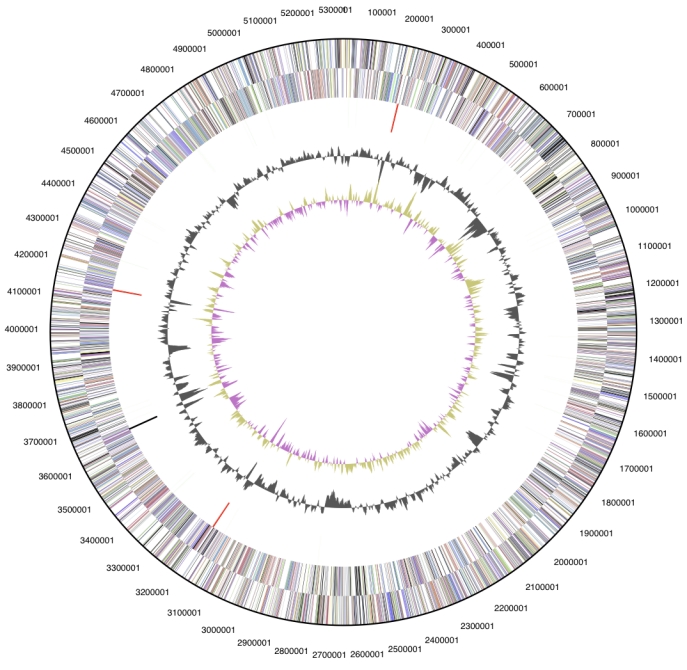
Graphical circular map of the genome. From outside to the center: Genes on forward strand (color by COG categories), Genes on reverse strand (color by COG categories), RNA genes (tRNAs green, rRNAs red, other RNAs black), GC content, GC skew.

The genome is 5,322,497 bp long and comprises one main chromosome with a 74.0% GC content (Table 3 and Figure 3). Of the 5,219 genes predicted 5,161 were protein coding genes, and 58 RNAs. In addition, 350 pseudogenes were also identified. The majority of the protein-coding genes (69.8%) were assigned with a putative function while those remaining were annotated as hypothetical proteins. The distribution of genes into COGs functional categories is presented in Table 4.

**Table 3:** Genome Statistics

**Attribute**	**Value**	**% of Total**
Genome size (bp)	5,322,497	100.00%
DNA coding region (bp)	4,756,139	89.36%
DNA G+C content (bp)	3,937,802	73.98%
Number of replicons	1	
Extrachromosomal elements	0	
Total genes	5,219	100.00%
RNA genes	58	1.11%
rRNA operons	3	
Protein-coding genes	5,161	98.89%
Pseudo genes	350	6,71%
Genes with function prediction	3,640	69.75%
Genes in paralog clusters	896	17.17%
Genes assigned to COGs	3,408	65.30%
Genes assigned Pfam domains	3,584	68.67%
Genes with signal peptides	793	15.19%
Genes with transmembrane helices	1,105	21.17%
CRISPR repeats	0	

**Table 4:** Number of genes associated with the general COG functional categories

**Code**	**Value**	**%age**	**Description**
J	166	3.2	Translation, ribosomal structure and biogenesis
A	1	0.0	RNA processing and modification
K	309	6.0	Transcription
L	196	3.8	Replication, recombination and repair
B	1	0.0	Chromatin structure and dynamics
D	27	0.5	Cell cycle control, mitosis and meiosis
Y	0	0.0	Nuclear structure
V	51	1.0	Defense mechanisms
T	242	4.7	Signal transduction mechanisms
M	213	4.1	Cell wall/membrane biogenesis
N	43	0.8	Cell motility
Z	0	0.0	Cytoskeleton
W	0	0.0	Extracellular structures
U	52	1.0	Intracellular trafficking and secretion
O	96	1.9	Posttranslational modification, protein turnover, chaperones
C	277	5.4	Energy production and conversion
G	267	5.2	Carbohydrate transport and metabolism
E	313	6.1	Amino acid transport and metabolism
F	87	1.7	Nucleotide transport and metabolism
H	180	3.5	Coenzyme transport and metabolism
I	188	3.6	Lipid transport and metabolism
P	164	3.2	Inorganic ion transport and metabolism
Q	127	2.5	Secondary metabolites biosynthesis, transport and catabolism
R	552	10.7	General function prediction only
S	295	5.7	Function unknown
-	1,811	35.1	Not in COGs

## Comparison with closest related genomes

Table 5 provides an overall comparison of the genomes of *G. obscurus* strain G-20^T^ with the closest available genomes, that is, *Acidothermus cellulolyticus* 11B^T^, *Frankia alni* ACN14A and *N. multipartita* Y-104^T^. The total length of (non-overlapping) high-scoring segment pairs (HSPs) and the number of identical base pairs within these HSPs were determined using the GGDC web server [42] by directly applying NCBI Blastn to the genomes represented as nucleotide sequences [43]. Number and proportion of shared homologs were determined using the 'Phylogenetic Profiler' function of the IMG (http://img.jgi.doe.gov/er) system [41] using default values. While the relative order of 16S rRNA difference does not correspond to the genomic similarities, the four genome-based measures uniformly indicate that *N. multipartita* Y-104^T^ possesses the genome most similar to the one of *G. obscurus* G-20^T^, followed by *F. alni* ACN14A and *A. cellulolyticus* 11B^T^.

**Table 5:** Percent-wise 16S rRNA sequence divergence ^1^

	**16S rRNA**	**GGD formula 1**	**GGD formula 2**	**GGD formula 3**	**Number of** **shared homologs**	**%age**
*A. cellulolyticus* 11B^T^ (NC_008578)	6.45%	0.930	0.231	0.946	2,309	44.7%
*F. alni* ACN14A (NC_008278)	5.81%	0.915	0.212	0.933	3,124	60.5%
*N. multipartita* Y-104^T^ (NC_013235)	6.76%	0.886	0.212	0.910	3,300	63.9%

^1^Percent-wise 16S rRNA sequence divergence compared to genomic similarity for the three closest available genomes to *G. obscurus* strain G-20^T^. GGD formulas: formula 1, length of sequence fragments not in HSPs per average total genome length; formula 2, number of non-identical bases per total HSP length; formula 3, number of non-identical bases within HSPs per average total genome length.

## References

[1] Luedemann GM *Geodermatophilus*, a new genus of the *Dermatophilaceae* (*Actinomycetales*). J Bacteriol 1968; 96:1848-1858 5726312 10.1128/jb.96.5.1848-1858.1968 PMC315248

[2] Normand P *Geodermatophilaceae* fam. nov., a formal description. Int J Syst Evol Microbiol 2006; 56:2277-2278 10.1099/ijs.0.64298-0 17012547

[3] Gordon MA The genus *Dermatophilus*. J Bacteriol 1964; 88:509-522 14203370 10.1128/jb.88.2.509-522.1964 PMC277328

[4] Stackebrandt E Kroppenstedt RM Fowler VJ A phylogenetic analysis of the family *Dermatophilaceae*. J Gen Microbiol 1983; 129:1831-1838 6195302 10.1099/00221287-129-6-1831

[5] Hahn D Lechevalier MP Fischer A Stackebrandt E Evidence for a close phylogenetic relationship between members of the genera *Frankia, Geodermatophilus* and *Blastococcus* and emendation of the family *Frankiaceae.* Syst Appl Microbiol 1989; 11:236-242

[6] Normand P Orso S Cournoyer B Jeannin P Chapelon, Dawson J, Evtushenko L, Misra AK. Molecular phylogeny of the genus *Frankia* and related genera and emendation of the family *Frankiaceae*. Int J Syst Bacteriol 1996; 46:1-9 10.1099/00207713-46-1-1 8573482

[7] Garrity GM, Lilburn TG, Cole JR, Harrison SH, Euzéby J, Tindall BJ. Taxonomic outline of the *Bacteria* and *Archaea*, Release 7.7 March 6, 2007. Part 10 - The *Bacteria*: Phylum *Actinobacteria*: Class "*Actinobacteria*". http://www.taxonomicoutline.org/index.php/toba/article/view/187/219

[8] Eppard M Krumbein WE Koch C Rhiel E Staley JT Stackebrandt E Morphological, physiological, and molecular characterization of actinomycetes isolated from dry soil, rocks, and monument surfaces. Arch Microbiol 1996; 166:12-22 10.1007/s002030050350 8661940

[9] Salazar O Valverde A Genilloud O Real-Time PCR for the detection and quantification of *Geodermatophilaceae* from stone samples and identification of new members of the genus *Blastococcus*. Appl Environ Microbiol 2006; 72:346-352 10.1128/AEM.72.1.346-352.2006 16391063 PMC1352205

[10] Hungate B Danin A Pellerin NB Stemmler J Kjellander P Adams JB Staley JT Characterization of manganese-oxidizing (MnII->MnIV) bacteria from Negev Desert rock varnish: implications in desert varnish formation. Can J Microbiol 1987; 33:939-943

[11] Kudukhashvili PG Gurielidze MA Pataraya DT Study of the lytic activities of actinomycetes isolated from different soils in Georgia. Appl Biochem Microbiol 2001; 37:251-252 10.1023/A:1010272900216 11443896

[12] Kuhlman KR Allenbach LB Ball CL Fusco WG La Duc MT Kuhlman GM Anderson RC Stuecker T Erickson IK Benardini J Enumeration, isolation, and characterization of ultraviolet (UV-C) resistant bacteria from rock varnish in the Whipple Mountains, California. Icarus 2005; 174:585-595 10.1016/j.icarus.2004.11.022

[13] Urzì C Brusetti L Salamone P Sorlini C Stackebrandt E Daffonchio D Biodiversity of *Geodermatophilaceae* isolated from altered stones and monuments in the Mediterranean basin. Environ Microbiol 2001; 3:471-479 10.1046/j.1462-2920.2001.00217.x 11553237

[14] Ishiguro EE Fletcher DW Characterization of *Geodermatophilus* strains isolated from high altitude Mount Everest soils. Mikrobiologika 1975; 12:99-108

[15] Ishiguro EE Wolfe RS Control of morphogenesis in *Geodermatophilus*: ultrastructural studies. J Bacteriol 1970; 104:566-580 5473909 10.1128/jb.104.1.566-580.1970 PMC248243

[16] Field D Garrity G Gray T Morrison N Selengut J Sterk P Tatusova T Thomson N Allen MJ Angiuoli SV The minimum information about a genome sequence (MIGS) specification. Nat Biotechnol 2008; 26:541-547 10.1038/nbt1360 18464787 PMC2409278

[17] Woese CR Kandler O Wheelis ML Towards a natural system of organisms: proposal for the domains *Archaea, Bacteria*, and *Eucarya.* Proc Natl Acad Sci USA 1990; 87:4576-4579 10.1073/pnas.87.12.4576 2112744 PMC54159

[18] Garrity GM, Holt JG. The Road Map to the Manual. *In:* Garrity GM, Boone DR, Castenholz RW (eds), *Bergey's Manual of Systematic Bacteriology*, Second Edition, Springer, New York, 2001, p. 119-169.

[19] Stackebrandt E rainey FA Ward-Rainey NL Proposal for a new hierarchic classification system, *Actinobacteria* classis nov. Int J Syst Bacteriol 1997; 47:479-491 10.1099/00207713-47-2-479

[20] O'Nuallain B Wetzel R Conformational Abs recognizing a generic amyloid fibril epitope. Proc Natl Acad Sci USA 2002; 99:1485-1490 10.1073/pnas.022662599 11818542 PMC122217

[21] Classification of *Bacteria* and *Archaea* in risk groups www.baua.de TRBA 466.

[22] Ashburner M Ball CA Blake JA Botstein D Butler H Cherry JM Davis AP Dolinski K Dwight SS Eppig JT Gene Ontology: tool for the unification of biology. Nat Genet 2000; 25:25-29 10.1038/75556 10802651 PMC3037419

[23] Mevs U Stackebrandt E Schumann P Gallikowski C Hirsch P *Modestobacter multiseptatus* gen. nov., sp. nov., a budding actinomycete from soils of the Asgard Range (Transantarctic Mountains). Int J Syst Evol Microbiol 2000; 50:337-346 10826821 10.1099/00207713-50-1-337

[24] Jordal PB Dueholm MS Larsen P Petersen SV Enghild JJ Christiansen G Hojrup P Nielsen PH Otzen DE Widespread abundance of functional bacterial amyloid in mycolata and other gram-positive bacteria. Appl Environ Microbiol 2009; 75:4101-4110 10.1128/AEM.02107-08 19395568 PMC2698375

[25] Venter JC Remington K Heidelberg JF Halpern AL Rusch D Eisen JA Wu D Paulsen I Nelson KE Nelson W Environmental genome shotgun sequencing of the Sargasso Sea. Science 2004; 304:66-74 10.1126/science.1093857 15001713

[26] Castresana J Selection of conserved blocks from multiple alignments for their use in phylogenetic analysis. Mol Biol Evol 2000; 17:540-552 10742046 10.1093/oxfordjournals.molbev.a026334

[27] Lee C Grasso C Sharlow MF Multiple sequence alignment using partial order graphs. Bioinformatics 2002; 18:452-464 10.1093/bioinformatics/18.3.452 11934745

[28] Stamatakis A Hoover P Rougemont J A Rapid Bootstrap Algorithm for the RAxML Web Servers. Syst Biol 2008; 57:758-771 10.1080/10635150802429642 18853362

[29] Pattengale ND Alipour M Bininda-Emonds ORP Moret BME Stamatakis A How Many Bootstrap Replicates Are Necessary? Lect Notes Comput Sci 2009; 5541:184-200 10.1007/978-3-642-02008-7_13 20377449

[30] Liolios K Chen IM Mavromatis K Tavernarakis N Hugenholtz P Markowitz VM Kyrpides NC The Genomes On Line Database (GOLD) in 2009: status of genomic and metagenomic projects and their associated metadata. Nucleic Acids Res 2010; 38:D346-D354 10.1093/nar/gkp848 19914934 PMC2808860

[31] Tice H Mayilraj S Sims D Lapidus A Nolan M Lucas S Glavina Del Rio T Copeland A Cheng JF Meincke L Complete genome sequence of *Nakamurella multipartita* type strain (Y-104^T^). Stand Genomic Sci 2010; (this issue). 10.4056/sigs.721316 PMC3035273 21304699

[32] Normand P Lapierre P Tisa LS Gogarten JP Alloisio N Bagnarol E Bassi CA Berry AM Bickhart DM Choisne N Cenome characteristics of facultatively symbiontic *Frankia* sp. strains reflect host range and host plant biogeography. Genome Res 2007; 17:7-15 10.1101/gr.5798407 17151343 PMC1716269

[33] Mirza MS Janse JD Hahn D Akkermans ADL Identification of atypical *Frankia* strains by fatty acid analysis. FEMS Microbiol Lett 1991; 83:91-98 10.1111/j.1574-6968.1991.tb04395.x

[34] Wink JM. Compendium of *Actinobacteria* http://www.gbif-prokarya.de/microorganisms/wink_pdf/DSM43160.pdf 2009.

[35] Lechevalier MP Lechevalier H Chemical composition as a criterion in the classification of aerobic actinomycetes. Int J Syst Bacteriol 1970; 20:435-443 10.1099/00207713-20-4-435

[36] List of growth media used at DSMZ: http://www.dsmz.de/microorganisms/media_list.php

[37] Wu D Hugenholtz P Mavromatis K Pukall R Dalin E Ivanova N Kunin V Goodwin L Wu M Tindall BJ A phylogeny-driven genomic en-cyclopedia of *Bacteria* and *Archaea*. Nature 2009; 462:1056-1060 10.1038/nature08656 20033048 PMC3073058

[38] Sims D Brettin T Detter J Han C Lapidus A Copeland A Glavina Del Rio T Nolan M Chen F Lucas S Complete genome sequence of *Kytococcus sedentarius* type strain (541^T^). Stand Genomic Sci 2009; 1:12-20 10.4056/sigs.761 PMC3035214 21304632

[39] Hyatt D Chen GL Locascio PF Land ML Larimer FW Hauser LJ Prodigal Prokaryotic Dynamic Programming Genefinding Algorithm. BMC Bioinformatics 2010; 11:119 10.1186/1471-2105-11-119 20211023 PMC2848648

[40] Pati A Ivanova N Mikhailova N Ovchinikova G Hooper SD Lykidis A Kyrpides NC GenePRIMP: A Gene Prediction Improvement Pipeline for microbial genomes. Nat Methods (In press). 10.1038/nmeth.1457 20436475

[41] Markowitz VM Ivanova NN Chen IMA Chu K Kyrpides NC IMG ER: a system for microbial genome annotation expert review and curation. Bioinformatics 2009; 25:2271-2278 10.1093/bioinformatics/btp393 19561336

[42] Auch AF Klenk HP Göker M Standard operating procedure for calculating genome-to-genome distances based on high-scoring sequence pairs. Stand Genomic Sci 2010; 2:142-148 10.4056/sigs.541628 PMC3035261 21304686

[43] Auch AF von Jan M Klenk HP Göker M Digital DNA-DNA hybridization for microbial species delineation by means of genome-to-genome sequence comparison. Stand Genomic Sci 2010; 2:117-134 10.4056/sigs.531120 PMC3035253 21304684

